# Time delay induced transition of gene switch and stochastic resonance in a genetic transcriptional regulatory model

**DOI:** 10.1186/1752-0509-6-S1-S9

**Published:** 2012-07-16

**Authors:** Canjun Wang, Ming Yi, Keli Yang, Lijian Yang

**Affiliations:** 1 Nonlinear Research Institute, Baoji University of Arts and Sciences, Baoji 721016, China; 2 Wuhan Institute of Physics and Mathematics, Chinese Academy of Sciences, Wuhan 430071, China; 3 National Center for Mathematics and Interdisciplinary Sciences, Chinese Academy of Sciences, Beijing 100190, China; 4 Department of Physics and Institute of Biophysics, Huazhong Normal University, Wuhan 430071, China

## Abstract

**Background:**

Noise, nonlinear interactions, positive and negative feedbacks within signaling pathways, time delays, protein oligomerization, and crosstalk between different pathways are main characters in the regulatory of gene expression. However, only a single noise source or only delay time in the deterministic model is considered in the gene transcriptional regulatory system in previous researches. The combined effects of correlated noise and time delays on the gene regulatory model still remain not to be fully understood.

**Results:**

The roles of time delay on gene switch and stochastic resonance are systematically explored based on a famous gene transcriptional regulatory model subject to correlated noise. Two cases, including linear time delay appearing in the degradation process (case I) and nonlinear time delay appearing in the synthesis process (case II) are considered, respectively. For case I: Our theoretical results show that time delay can induce gene switch, i.e., the TF-A monomer concentration shifts from the high concentration state to the low concentration state ("on"*→*"off"). With increasing the time delay, the transition from "on" to "off" state can be further accelerated. Moreover, it is found that the stochastic resonance can be enhanced by both the time delay and correlated noise intensity. However, the additive noise original from the synthesis rate restrains the stochastic resonance. It is also very interesting that a resonance bi-peaks structure appears under large additive noise intensity. The theoretical results by using small-delay time-approximation approach are consistent well with our numerical simulation. For case II: Our numerical simulation results show that time delay can also induce the gene switch, however different with case I, the TF-A monomer concentration shifts from the low concentration state to the high concentration state ("off"*→*"on"). With increasing time delay, the transition from "on" to "off" state can be further enhanced. Moreover, it is found that the stochastic resonance can be weaken by the time delay.

**Conclusions:**

The stochastic delay dynamic approach can identify key physiological control parameters to which the behavior of special genetic regulatory systems is particularly sensitive. Such parameters might provide targets for pharmacological intervention. Thus, it would be highly interesting to investigate if similar experimental techniques could be used to bring out the delay-induced switch and stochastic resonance in the stochastic gene transcriptional regulatory process.

## Background

In recent years, a plenty of researches show that noises play a positive role in many fields. Many novel phenomena are found, such as noise induced transition [1-3], reentrance phenomena [4,5], stochastic resonance [6,7], noise enhanced stability [8,9], current reveal [10-12], noise enhanced coherence resonance [13,14], and so on. On the other hand, in many cases, the delay reflects transmission times related to the transport of matter, energy, and information through the system. Understanding the behavior of time-delayed dynamical systems is a first step in improving the knowledge of memory in general, whose analysis is especially important in medicine, biology and control theory. Recently, the combined effects of noises and time delays have been the subject of increased interest. In the field of pure statistical physics, the bistable systems with noise and time delay simultaneous have been investigated in detail [15-17]. Brownian motor with time-delayed feedback is studied by Wu [18]. The effect of time delay on feedback control of a flashing ratchet has been also investigated [19]. The integration of noise and time delay completely suppresses the population explosion in a mutualism [20]. Effects of time delays and noises in competitive systems have been investigated [21]. These results implicated that the combination of noise and time delay could provide an efficient tool for understanding real systems.

Regulation of gene expression by signals outside and inside the cell plays important roles in many biological processes. As the basic principles of genetic regulation have been characterized, it has become increasingly evident that nonlinear interactions, positive and negative feedback within signaling pathways, time delays, protein oligomerization, and crosstalk between different pathways need to be considered for understanding genetic regulation [22-28]. Smolen *et al*. have introduced a simple genetic regulatory model that incorporates known features of genetic regulatory using an explicitly mathematical dynamic systems approach [22,23]. The simplest model manifested multiple stable steady states, and brief perturbations could switch the model between these states. Moreover, the effects of macromolecular transport and stochastic fluctuations on dynamics of genetic regulatory systems are investigated. Liu *et al*. [25] have studied the effects of the correlation between the noise of the decomposed rate *k_d _*and the noise of the synthesis rate *R_bas_*. They found that a successive switch process (i.e., "on"*→*"off"*→*"on", which we call the reentrance transition or twice switch) occurs with increasing the noise intensities, and a critical noise intensity exists at which the mean first passage time of the switch process is the largest. The effect of the color cross-correlated on the switch is investigated [26]. Wang [27]* et al*. also have investigated the effects of delay time, which is the time required for movement of TF-A protein to the nucleus. Their results showed that the delay time restrains the transition from the low concentration state to the high concentration state. However, these studies only consider single noise source, in particular, the delay-induced switch-like behaviors has not been explored yet. In addition, in this case the delay time appears in both deterministic and fluctuating forces simultaneously, hence it is very difficult to study from a view of theoretical analysis.

Stochastic resonance (SR), which was originally discovered by Benzi and Nicolis [29,30] in the context of modeling the switch of the Earth's climate between ice ages and periods of relative warmth, is an important aspect in many scientific fields, which has been investigated extensively due to its potential applications from both the theoretical and experimental points of view. SR is a common case and generic enough to be observable in a large variety of nonlinear dynamical systems, including the occurrence of SR in physical systems, biological system, ecological system, laser system, etc. In the biophysics field, the study of SR phenomenon has turned into a forward subject. The SR phenomenon and its applications were extensively found. Such as, noise enhancement of information transfer in crayfish mechanoreceptors by SR [7]. SR can be used as a measuring tool to quantify the ability of the human brain to interpret noise contaminated visual patterns [31] and appears in an anti-tumor system modulated by a seasonal external field [32]. Oscillation and noise determine signal transduction in shark multimodal sensory cells [33]. The gene expression can be regulated by signals from outside and within the cell. Thereby, in the gene transcriptional regulatory process, the external environmental factors, such as the electromagnetic field on the earth, the solar terms and seasonal variation, are the common features. This means that the transcriptional regulatory of gene should have a periodic form. In this case, the bistability, noise and the signal exist simultaneously, so the combined effects of noises and delay time on the SR should be investigated.

We would like to emphasize that the combined effects of correlated noise and time delay on dynamical behaviors of gene regulatory network are rarely investigated. In this article, the statistical properties of gene switch and stochastic resonance induced by time delay in two different cases (i.e., linear time delay case and nonlinear time delay case) are explored. Our investigation is a significant try forward understanding the basic mechanisms of the delay induced gene switch and stochastic resonance in realistic yet complex organisms from a view of theory, and will motivate the further experimental research for gene network.

## Model

### Deterministic gene transcriptional regulatory model

**Figure 1:**
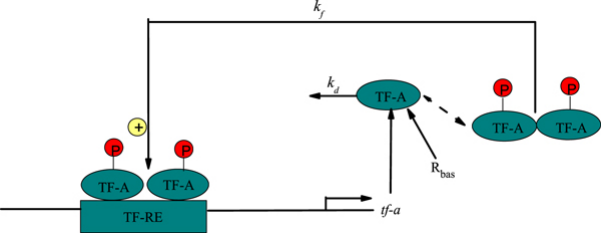
**Model of genetic regulation with a positive autoregulatory feedback loop**. The transcription factor activator (TF-A) activates transcription with a maximal rate *k_f _*when phosphorylated (P) and binds as a dimer to specific responsive-element DNA sequences (TF-REs). TF-A is degraded with rate *k_d _*and synthesized with rate *R_bas_*.

To examine the capability of genetic regulatory systems for complex dynamic activity, Smolen *et al*. [22] have developed simple kinetic models that incorporate known features of these systems. These features include autoregulation and stimulus-dependent phosphorylation of transcription factors (TFs), dimerization of TFs, crosstalk, and feedback. The simplest kinetic model of genetic regulation can be described by Figure 1. A single TF-A is considered as part of a pathway mediating a cellular response to a stimulus. The TF forms a homodimer that can bind to responsive elements (TF-REs). The TF-A gene incorporates a TF-RE, and when homodimers bind to this element, TF-A transcription is increased. Binding to the TF-REs is independent of dimer phosphorylation. Only phosphorylated dimers can activate transcription. The fraction of dimers phosphorylated is dependent on the activity of kinases and phosphatases whose activity can be regulated by external signals. Thus, this model incorporates both signal-activated transcription and positive feedback on the rate of TF synthesis. It is assumed that the transcription rate saturates with TF-A dimer concentration to maximal rate *k_f_*, which is proportional to TF-A phosphorylation. At negligible dimmer concentration, the synthesis rate is *R_bas_*. TF-A is eliminated with a rate constant *k_d_*, binding processes are considered comparatively rapid, so the concentration of dimmer is proportional to the square of TF-A monomer concentration *x*. These simplifications give a model with a single ordinary differential equation for the concentration of the TF-A:

(1) $$\frac{dx\left(t\right)}{dt}=\frac{k_{f}x^{2}\left(t\right)}{x^{2}\left(t\right)+K_{d}}-k_{d}x\left(t\right)+R_{bas},$$

where *K_d _*is the dissociation concentration of the TF-A dimer from TF-REs. Under the following condition of parameters:

(2) $${\left[-{\left(\frac{k_{f}+R_{bas}}{3k_{d}}\right)}^{3}+\frac{K_{d}\left(k_{f}+R_{bas}\right)}{6k_{d}}-\frac{K_{d}R_{bas}}{2k_{d}}\right]}^{2}+{\left[\frac{K_{d}}{3}-{\left(\frac{k_{f}+R_{bas}}{3k_{d}}\right)}^{2}\right]}^{3}<0.$$

The potential function corresponding to Eq.(1) is

(3) $$U_{0}\left(x\right)=k_{f}\sqrt{K_{d}}\text{arctan}\left(\frac{x}{\sqrt{K_{d}}}\right)+\frac{k_{d}}{2}x^{2}-\left(R_{bas}+k_{f}\right)x.$$

Two stable steady states are presented as $x_{+}=2\sqrt{-p/3}cos\left(\theta \right)+\left(R_{bas}+k_{f}\right)/\left(3k_{d}\right)$ and $x_{-}=2\sqrt{-p/3}cos\left(\theta +2\pi \text{/3}\right)+\left(R_{bas}+k_{f}\right)/\left(3k_{d}\right)$, respectively. One unstable steady state is $x_{u}=2\sqrt{-p/3}cos\left(\theta +4\pi \text{/3}\right)+\left(R_{bas}+k_{f}\right)/\left(3k_{d}\right)$, where *p *= *K_d _*- [(*R_bas _+ k_f_*)*/k_d_*]^2 ^/3, *q *= *K_d_*(*k*_*f *_- 2*R_bas_*)*/*(*3k_d_*) - 2[(*R*_*bas *_*+ k*_*f*_)/(*3k_d_*)]^3 ^and $\theta =arccos(-q/(2\sqrt{-p^{3}/27)}/3$

**Figure 2:**
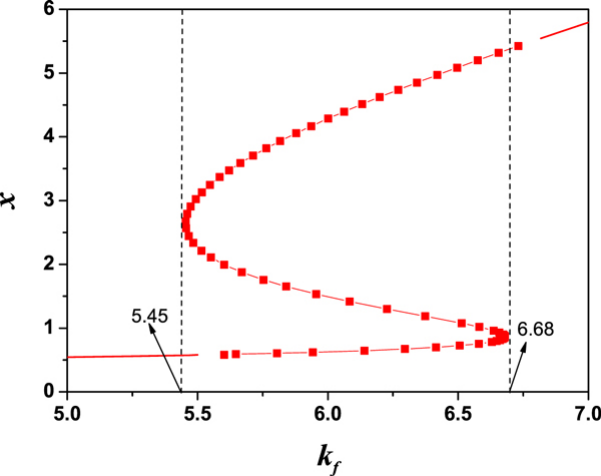
**Bifurcation plot for the steady state of TF-A on the control parameter of transcription rate *k_f_***. The system in the region (i.e., 5.45 *< k_f _<*6.68) exhibits bistability. The other parameters are fixed as dissociation constant of TF-A dimer from TF-REs *K_d _*= 10, the degradation constant *k_d _*= 1, and the basal rate of TF-A synthesis *R_bas _*= 0.4.

**Figure 3:**
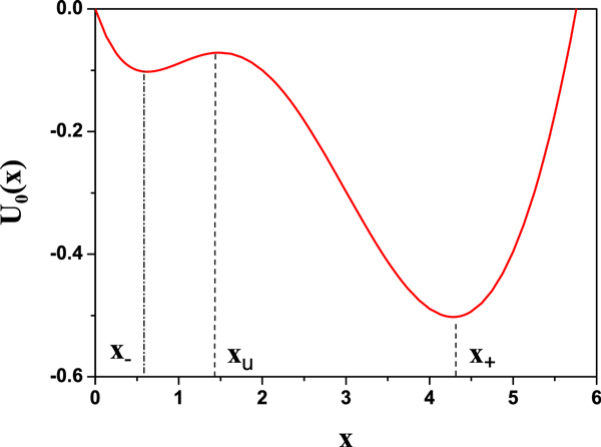
**The bistable potential of Eq.(3)**. The parameter values are *k_f _*= 6, *K_d _*= 10, *k_d _*= 1, and *R_bas _*= 0.4.

An interesting aspect of the model is that, based on the different initial conditions, the concentration of TF-A can be one of the two stable steady states. It is a bistable system for certain values of *k_f _*(i.e., 5.45 *< k_f _<*6.68) (see Figure 2). Bistability is a kind of important dynamical feature in biological systems, especially for the fate decision in some biological processes. In this article, our works are employed in the bistable region. When the parameter values are *k_f _*= 6, *K_d _*= 10, *k_d _*= 1 and *R_bas _*= 0.4, the stable steady states are *x*_ *≈*0.62685 and *x_+ _≈*4.28343, and the unstable steady state is *x_u _≈*1.48971 as shown in Figure 3[25].

### Stochastic model with correlated noise and time delay

Cells are intrinsically noisy biochemical reactors: low reactant numbers can lead to significant statistical fluctuations in molecule numbers and reaction rates [34]. It has been found that the stability against fluctuations is essential for the gene regulatory cascade controlling cell differentiation in a developing embryo [35]. Moreover, these fluctuations are intrinsic: they are determined by structure, reaction rates, and species concentrations of the underlying biochemical networks. So we should not only consider the deterministic model. Recently, some experiments also showed that *R_bas _*and *k_d _*are affected by the biochemical reactions, mutations and the concentrations of other proteins also fluctuate [36]. Therefore, it is reasonable to study the fluctuation effects on the gene transcriptional regulatory model. We consider the fluctuations both on the synthesis rate *R_bas _*and the rate constant *k*_*d*_. Namely *R_bas _→ R_bas _+ η*(*t*) and *k*_*d *_*→ k*_*d *_+ ξ(*t*). The two independent noise ξ(*t*) and *η*(*t*) may have a common source, thereby the correlation between them should be taken into our model. The stochastic differential equation (Langevin equation) corresponding to this bistable gene model is given:

(4) $$\frac{dx\left(t\right)}{dt}=\frac{k_{f}x^{2}\left(t\right)}{x^{2}\left(t\right)+K_{d}}-\left(k_{d}+\xi \left(t\right)\right)x\left(t\right)+R_{bas}+\eta \left(t\right),$$

where ξ(*t*) and *η*(*t*) are the Gaussian white noise with the following statistical properties:

(5) ⟨ξ(t)⟩=⟨η(t)⟩=0,

(6) $$\left\langle \xi \left(t\right)\xi \left(t^{{}^{\prime }}\right)\right\rangle =2D\delta \left(t-t^{{}^{\prime }}\right),$$

(7) $$\left\langle \eta \left(t\right)\eta \left(t^{{}^{\prime }}\right)\right\rangle =2\alpha \delta \left(t-t^{{}^{\prime }}\right),$$

(8) $$\left\langle \xi \left(t\right)\eta \left(t^{{}^{\prime }}\right)\right\rangle =\left\langle \eta \left(t\right)\xi \left(t^{{}^{\prime }}\right)\right\rangle =2\lambda \sqrt{\alpha D}\delta \left(t-t^{{}^{\prime }}\right).$$

Where *D *and *α *denote the multiplicative and additive noise intensities, respectively, and λ represents the coupling strength between the two noise terms (i.e., correlated intensity).

In order to more exactly predict the dynamics of the genetic regulation model, it is necessary to consider macromolecular transport in these biochemical reactions. Transport can be diffusive or active, and in some cases a time delay may suffice to model active transport. Smolen *et al. *[22,23] have considered that the binding processes of gene transcriptional regulatory are comparatively rapid, and would probably not be reasonable for overall cellular nuclear concentration of TF-A, because the equilibration time would be on the order of the degradation time for TF-A protein. However, a short time scale for equilibration is more likely for nuclear concentration of TF-A. This is because the rate constants *k_f _*and *k_d _*include implicitly entrance and exit of TF-A protein from the relatively small nuclear volume and are thus larger than those governing the dynamics of overall cellular concentration of TF-A. Therefore, the time delay should be considered in this model. This delay time appears between any change in the level of nuclear TF-A and the appearance in the nucleus of TF-A synthesized and degrading process in response to that change.

### Case I: Linear time delay appearing in the degradation process

**Figure 4:**
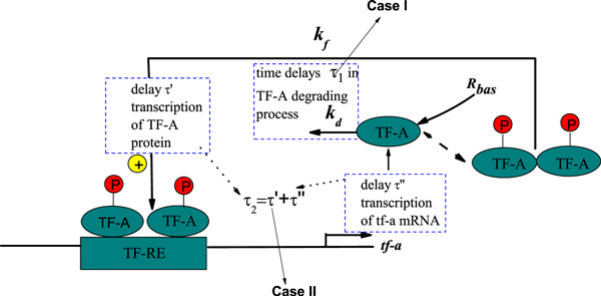
**Model of genetic regulation with a positive autoregulatory feedback loop and time delays**. **Case I: **time delay *τ*_1 _in the degradation process of TF-A; **Case II:** time delay *τ*_2 _= *τ' + τ*″, with *τ' *the time taken for the transcription of tf-a mRNA and its movement to translation and *τ*″ the time required for movement of TF-A protein to the nucleus.

First, we consider the local time delay due to the degradation of TF-A in the nucleus. The simplest kinetic model of genetic regulation with the local time delay is described by **Case I **in Figure 4. The time delay τ_1 _appearing in the TF-A degradation process can affect the TF-A monomer concentration *x*(*t*). Therefore, (*k_d _+ ξ*(*t*))*x*(*t*) can be written as (*k_d _+ ξ*(*t*))*x*(*t - τ*_1_), and Eq. (4) is further rewritten:

(9) $$\frac{dx\left(t\right)}{dt}=\frac{k_{f}x^{2}\left(t\right)}{x^{2}\left(t\right)+K_{d}}-k_{d}x\left(t-\tau_{1}\right)+R_{bas}-x\left(t-\tau_{1}\right)\xi \left(t\right)+\eta \left(t\right).$$

where the *τ*_1 _(time delay) previous to the time when *dx/dt *is computed. Because *k_d_x*(*t - τ*) is dependent linearly on the TF-A monomer concentration, for simplicity, we call this form of time delay as linear time delay. In addition, only small time delay is investigated in Case I since the theoretical approximation methods below are applicable for the small delay time.

### Case II: Nonlinear time delay appearing in the synthesis process

Second, the rate constant *k_f _*includes implicitly entrance and exit of TF-A protein from the relatively small nuclear volume, thus larger than those governing the dynamics of overall cellular [TF-A]. We may consider the local time delay appearing in the synthesis process. The model incorporates a time delay *τ*_2 _= *τ' + τ*″, where *τ' *is the time taken for the transcription of tf-a mRNA and its movement to translation, and *τ*″ is the time required for movement of TF-A protein to the nucleus. Namely, the local time delay is introduced into the Hill function. The simplest kinetic model of genetic regulation with time delay appearing in Hill function is presented by Case II in Figure 4.

Then, $\frac{k_{f}x^{2}\left(t\right)}{x^{2}\left(t\right)+K_{d}}\to \frac{k_{f}x^{2}\left(t-\tau_{2}\right)}{x^{2}\left(t-\tau_{2}\right)+\tau K_{d}}$, and *E*q. (4) can be rewritten:

(10) $$\frac{dx\left(t\right)}{dt}=\frac{k_{f}x^{2}\left(t-\tau_{\text{2}}\right)}{x^{2}\left(t-\tau_{\text{2}}\right)+K_{d}}-k_{d}x\left(t\right)+R_{bas}-x\left(t\right)\xi \left(t\right)+\eta \left(t\right).$$

where the first term on the right side is evaluated at a time *τ*_2 _(delay time) previous to the time when *dx/dt *is computed, and is nonlinear time-delayed, and the delay time does not appear in the stochastic force. Because $\frac{k_{f}x^{2}\left(t-\tau_{\text{2}}\right)}{x^{2}\left(t-\tau_{\text{2}}\right)+K_{d}}$ is dependent nonlinearly on the TF-A monomer concentration, for simplicity, we regard this case as nonlinear time delay case.

Below, the statistics properties of our theoretical model subjected to correlated noise and time delay are explored in the two different cases (i.e., linear time delay case and nonlinear time delay case). Considering the difficulties in theoretical analysis, we will investigate the two different time delays in the gene model, respectively.

## Methods and results

### Results for case I

#### Steady-state probability distribution

The small time delay approximation of the probability density approach is employed [37,38]. Substituting $x_{\tau_{\text{1}}}$ for *x*(*t - τ*_1_) in Eq.(9), we obtain

(11) $$\frac{dx\left(t\right)}{dt}=h_{eff}\left(x\left(t\right)\right)+g_{eff}\left(x\left(t\right)\right)\xi \left(t\right)+\eta \left(t\right),$$

where

(12) $$\begin{array}{llll} h_{eff}\left(x\right) & =\int_{-\infty }^{+\infty }\left(\frac{k_{f}x^{2}}{x^{2}+K_{d}}-k_{d}x_{\tau_{\text{1}}}+R_{bas}\right)P_{d}\left(x_{\tau_{\text{1}}},t-\tau_{\text{1}};x,t\right)dx_{\tau_{\text{1}}}\; & & \;\\ & =\left(1+\tau_{\text{1}}\right)\left(\frac{k_{f}x^{2}}{x^{2}+K_{d}}-k_{d}x+R_{bas}\right).\; & & \;\\ & \; & \end{array}$$

(13) $$g_{eff}\left(x\right)=\int_{-\infty }^{+\infty }\left(-x_{\tau_{\text{1}}}\right)P_{s}\left(x_{\tau_{\text{1}}},t-\tau_{\text{1}};x,t\right)dx_{\tau_{\text{1}}}=-\left(1+\tau_{\text{1}}\right)x.$$

In Eq.(11)-(12), $P_{d}\left(x_{\tau_{\text{1}}},t-\tau_{\text{1}};x,t\right)$ and $P_{s}\left(x_{\tau_{\text{1}}},t-\tau_{\text{1}};x,t\right)$ denote the conditional distributions of *x*(*t*) in the deterministic part and stochastic part, respectively, which are given by [39]

(14) $$P_{d}\left(x_{\tau_{\text{1}}},t-\tau_{\text{1}};x,t\right)=\sqrt{\frac{1}{2\pi G^{2}\left(x,x\right)\tau_{\text{1}}}\text{exp}\left(-\frac{{\left[x_{\tau_{\text{1}}}-\left(x+h\left(x,x\right)\tau_{\text{1}}\right)\right]}^{2}}{2G^{2}\left(x,x\right)\tau_{\text{1}}}\right),}$$

(15) $$P_{s}\left(x_{\tau_{\text{1}}},t-\tau_{\text{1}};x,t\right)=\sqrt{\frac{1}{2\pi G^{2}\left(x,x\right)\tau_{\text{1}}}\text{exp}\left(-\frac{{\left[x_{\tau_{\text{1}}}-\left(x+g\left(x,x\right)\tau_{\text{1}}\right)\right]}^{2}}{2G^{2}\left(x,x\right)\tau_{\text{1}}}\right),}$$

where $h\left(x,x\right)=\frac{k_{f}x^{2}}{x^{2}\left(t\right)+K_{d}}-k_{d}x+R_{bas},g\left(x,x\right)=-x,G^{2}\left(x,x\right)=Dx^{2}-2\lambda \sqrt{D\alpha }x+\alpha$. Thus, the stochastic delayed differential equation can be approximately reduced to the ordinary stochastic equation. The non-Markovian process induced by the time delay can be converted into Markovian process. Meanwhile, Eq.(11) is equivalently transformed into a stochastic differential equation [2]

(16) $$\frac{dx\left(t\right)}{dt}=h_{eff}\left(x\left(t\right)\right)+G_{eff}\left(x\right)\varepsilon \left(t\right),$$

with

(17) $$\left\langle \varepsilon \left(t\right)\varepsilon \left(t^{{}^{\prime }}\right)\right\rangle =2\delta \left(t-t^{{}^{\prime }}\right),$$

(18) $$\begin{array}{llll} G_{eff}\left(x\right) & =\sqrt{Dg_{eff}{\left(x\right)}^{2}-2\lambda \sqrt{D\alpha }g_{eff}\left(x\right)+\alpha }\; & & \;\\ & =\sqrt{D{\left(1+\tau_{\text{1}}\right)}^{2}x^{2}-2\lambda \sqrt{D\alpha }\left(1+\tau_{\text{1}}\right)x+\alpha }.\; & & \;\\ & \; & \end{array}$$

In the steady-state regime (given by Eq.(2)) and under the constraint *x *> 0 (the TF-A monomer concentration *x*(*t*) is all higher than zero), the approximate delay Fokker-Planck equation corresponding to Eq.(16) is derived as

(19) $$\frac{\partial }{\partial t}P\left(x,t\right)=-\frac{\partial }{\partial x}A\left(x\right)P\left(x,t\right)+\frac{\partial^{2}}{\partial x^{2}}B\left(x\right)P\left(x,t\right).$$

where

(20) $$A\left(x\right)=h_{eff}\left(x\right)+G_{eff}\frac{dG_{eff}\left(x\right)}{dx},$$

(21) $$B\left(x\right)=G_{eff}^{2}\left(x\right).$$

The stationary probability distribution (SPD) corresponding to Eq. (19) is obtained

(22) $$\begin{array}{llll} P_{st}\left(x\right) & =\frac{N}{G_{eff}}\text{exp}\int_{0}^{x}\frac{h_{eff}\left(x^{{}^{\prime }}\right)}{B\left(x^{{}^{\prime }}\right)}dx^{{}^{\prime }},\; & & \;\\ & =\frac{N}{\sqrt{D{\left(1+\tau_{\text{1}}\right)}^{2}x^{2}-2\lambda \sqrt{D\alpha }\left(1+\tau_{\text{1}}\right)x+\alpha }}\text{exp}\left[\Phi \left(x\right)\right],\; & & \;\\ & \; & \end{array}$$

where *N *is a normalization constant, and Φ(*x*) is the generalized potential function following

(23) $$\begin{array}{llll} \Phi \left(x\right) & =\frac{1}{\gamma_{0}}[\gamma_{1}\text{ln}\left(x^{2}+k_{d}\right)+\gamma_{2}\text{arctan}\left(\frac{x}{\sqrt{k_{d}}}\right)+\gamma_{3}\text{ln}\left(Dn^{2}x^{2}-2mnx+\alpha \right)\; & & \;\\ & \;+\frac{\gamma_{4}}{\sqrt{D\alpha n^{2}-m^{2}n^{2}}}\text{arctan}\left(\frac{Dn^{2}x-mn}{D\alpha n^{2}-m^{2}n^{2}}\right)],\; & & \;\\ & \; & \end{array}$$

where

(24) $$\begin{array}{c} n=1+\tau_{\text{1}},\\ m=\lambda \sqrt{D\alpha },\\ \gamma_{\text{0}}=4m^{2}K_{d}n^{2}-2\alpha Dn^{2}K_{d}+K_{d}^{2}D^{2}n^{4}+\alpha^{2},\\ \gamma_{\text{1}}=-K_{d}k_{f}n^{2}m,\\ \gamma_{\text{2}}=K_{d}^{3/2}k_{f}Dn^{3}-n\sqrt{K_{d}}k_{f},\\ \gamma_{\text{3}}=n^{2}mK_{d}k_{f}-\frac{k_{d}\alpha^{2}}{2Dn}-\frac{2nm^{2}K_{d}k_{d}}{D_{3}}+n\alpha k_{d}K_{d}-\frac{Dn^{3}K_{d}^{2}k_{d}}{2},\\ \gamma_{\text{4}}=-\alpha K_{d}Dn^{3}k_{f}+n\alpha^{2}k_{f}+4n^{3}m^{2}R_{bas}k_{d}\\ \;\;-2Dn^{3}R_{bas}K_{d}\alpha +D^{2}n^{5}R_{bas}K_{d}^{2}+nR_{bas}\alpha^{2}\\ \;\;+2n^{3}m^{2}K_{d}k_{f}-mk_{d}\alpha^{2}-4n^{2}m^{3}K_{d}k_{d}\\ \;\;+2n^{2}m\alpha k_{d}K_{d}-n^{4}mDK_{d}^{2}k_{d}. \end{array}$$

**Figure 5:**
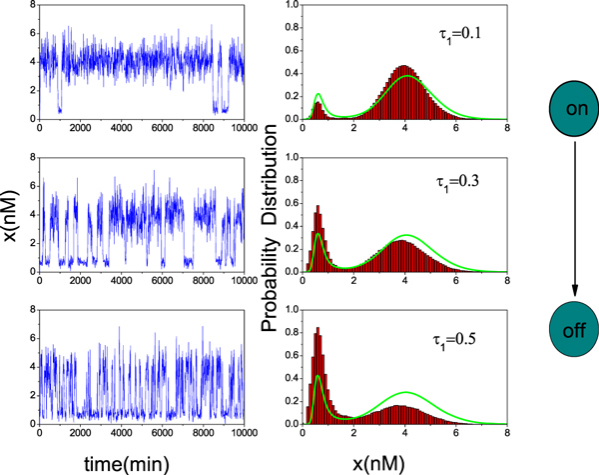
**Sample paths and probability distribution of *x(t) *for different delay time *τ*_1_**. From top to bottom *τ*_1 _= 0.1, 0.3, and 0.5. *α *= 0.01, D = 0.15 and *λ *= 0.3. The green curve on the right side is the SPD by using of Eq. (22). The other parameter values are the same as those in Figure 2.

In the bistable region, the time course of TF-A monomer concentration *x*(*t*) and the probability distribution are plotted for different delay time, as shown in Figure 5, respectively. These results are obtained by directly simulating the stochastic differential equation (9) and by using the theoretical formula (22), respectively. From Figure 5, it is clear that the TF-A monomer concentration *x *shifts from the high concentration state to the low concentration state with increasing the delay time *τ*_1_. If we regard the low concentration state as the "off" state and the high concentration state as the "on" state, the above result indicates that a switch process can be induced by the delay time. Figure 5 shows that the TF-A monomer concentration *x *concentrates on the high concentration state when the delay time is small, that is, we begin the switch in the "on" position by tuning the delay time to a very low value. However, increasing the delay time causes the low concentration state to become populated. It means that the concentration of TF-A monomer decreases, and a flipping of the switch to the "off" position occurs. Therefore, the delay time can be used as a control parameter for the switch process in the genetic regulatory system. The agreement between our theoretical and numerical results indicates that the approximation method seems to work quite well for the small delay time.

#### Mean value

In order to quantitatively investigate the stationary properties of the system, we introduce the moments of the variable *x *as

(25) $${\left\langle x^{n}\right\rangle }_{st}=\int_{0}^{+\infty }x^{n}P_{st}\left(x\right)dx.$$

The mean of the state variable *x *is

(26) $${\left\langle x\right\rangle }_{st}=\int_{0}^{+\infty }xP_{st}\left(x\right)dx.$$

**Figure 6:**
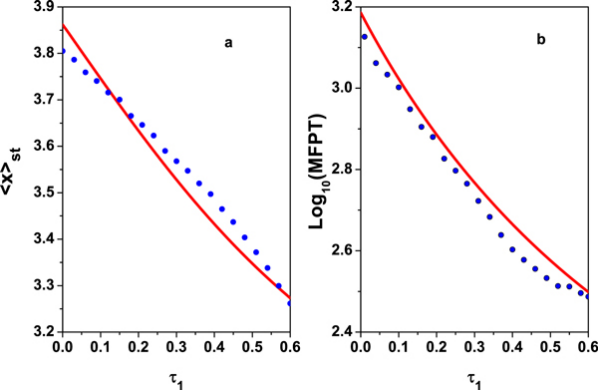
***< x >_st _*and MFPT are plotted as a function of delay time *τ*_1 _with *α *= 0.01, *D *= 0.015 and *λ *= 0.3**. The other parameter values are the same as those in Figure 2. The red sold line represents the theoretical results, and the blue dot line represents the numerical simulation results. (a)*< x >_st_; *(b) MFPT.

The theoretical and the numerical simulation results of 〈*x*〉_*st *_as a function of *τ*_1 _is plotted in Figure 6(a). Figure 6(a) shows that the 〈*x*〉_*st *_is decreased with increasing *τ*_1_. When *τ*_1 _is small, the TF-A monomer concentrates on the high concentration state. When *τ*_1 _is increased, the TF-A monomer concentrates on the low concentration state. Namely, for large *τ*_1_, it is more easy to be at the "off" state (the low concentration state). The effect is similar to the effect of *τ*_1 _on SPD shown in Figure 5. It also implicates that the time delay induces the gene transition from the "on" state to the "off" state.

#### Mean first passage time

For the delay time-induced switch, we will quantify the effects of delay time on the switch between the two stable steady states. When the system is stochastically bistable, a quantity of interest is the time from one state to the other state, which is often referred to as the first passage time. We consider the mean first passage time (MFPT). Here the MFPT of the process *x*(*t*) to reach the low concentration state *x*_(t) with initial condition *x*(*t *= 0)=*x_+ _*(the high concentration state) is provided by [40],

(27) $$T\left(x_{+}\to x_{-}\right)=\int_{x_{+}}^{x_{-}}\frac{dx}{B\left(x\right)P_{st}\left(x\right)}\int_{0}^{x}P_{st}\left(y\right)dy.$$

When the intensities of noises terms *D *are small enough compared with the energy barrier height ΔΦ(*x*) = Φ(*x*+) - Φ(*x_u_*), we can apply the steepest-descent approximation to Eq.(27). Hence *T *is simplified as following [41]

(28) $$T\left(x_{+}\to x_{u}\right)\approx \frac{2\pi }{\sqrt{\left|{U^{\prime \prime }}_{0}\left(x_{+}\right){U^{\prime \prime }}_{0}\left(x_{u}\right)\right|}}\text{exp}\left[\frac{\Phi \left(x_{u}\right)-\Phi \left(x_{+}\right)}{D}\right].$$

Here, the potential *U*_0_(*x*) is given by Eq.(3) and Φ(*x*) is given by Eq.(23).

By virtue of Eq.(28), the effects of *τ*_1 _on the MFPT can be analyzed. MFPT as a function of *τ*_1 _is plotted in Figure 6(b). It shows that MFPT decreases monotonously as *τ*_1 _increases. From the view point of physics, it means that the delay time can speed up the transition between the two steady states (low concentration state and high concentration state). Namely, the delay time can accelerate the transition of gene switch from "on" state to "off" state.

#### Effects of time delay on stochastic resonance

**Figure 7:**
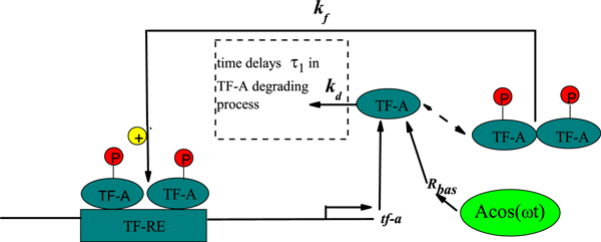
**Model of genetic regulation with a positive autoregulatory feedback loop, delay time and an additive signal *Acos*(Ω *t*)**.

In the gene transcriptional regulatory process, the external environmental factors, such as the electromagnetic field on the earth, the solar terms and seasonal variation, are the common features. This means that the transcript of gene should have a periodic form. For simplicity, a cosinoidal form *Acos*(*Ωt*) is adopted to model. The model is shown in Figure 7. If integrating the correlated noises, the delay time and the weak periodic signal, we can rewrite Eq.(9) as following

(29) $$\frac{dx\left(t\right)}{dt}=\frac{k_{f}x^{2}\left(t\right)}{x^{2}\left(t\right)+K_{d}}-k_{d}x\left(t-\tau_{\text{1}}\right)+R_{bas}-x\left(t-\tau_{\text{1}}\right)\xi \left(t\right)+\eta \left(t\right)+Acos\left(\Omega t\right),$$

where *ξ*(*t*) and *η*(*t*) are the Gaussian white noise, and their statistical properties are given by Eqs.(5)-(8). *A *is the amplitude of input periodic signal, Ω is the frequency, and *τ*_1 _is the delay time.

#### Signal to noise ratio

Making use of the small delay time approximation of the probability density approach and the stochastic equivalence method, the approximated delay Fokker-Planck equation of this model is given by

(30) $$\begin{array}{c} \frac{\partial P\left(x,t\right)}{\partial t}=-\frac{\partial }{\partial x}\left[\left(\left(1+\tau_{\text{1}}\right)\left(\frac{k_{f}x^{2}}{x^{2}+K_{d}}-k_{d}x+R_{bas}+Acos\left(\Omega t\right)\right)+D{\left(1+\tau_{\text{1}}\right)}^{2}x-\lambda \sqrt{D\alpha }\left(1+\tau_{\text{1}}\right)\right)P\left(x,t\right)\right]\\ \;+\frac{\partial^{2}}{\partial x^{2}}\left[\left(D{\left(1+\tau_{\text{1}}\right)}^{2}x^{2}-2\lambda \sqrt{D\alpha }\left(1+\tau_{\text{1}}\right)x+\alpha \right)P\left(x,t\right)\right]. \end{array}$$

Under the constraint *x *> 0 (the TF-A monomer concentration *x*(*t*) is always higher than zero in the bistable region satisfying Eq.(2), the quasi-steady-state distribution function *P_qst_*(*x, t*) can be derived from Eq.(30) in the adiabatic limit:

(31) $$P_{qst}\left(x,t\right)=\frac{N}{{\left(D{\left(1+\tau_{\text{1}}\right)}^{2}x^{2}-2\lambda \sqrt{D\alpha }\left(1+\tau_{\text{1}}\right)x+\alpha \right)}^{1/2}}\text{exp}\left[-\frac{\phi_{n}\left(x,t\right)}{D}\right],$$

where *N *is a normalization constant, *φ_n_*(*x, t*) is the generalized potential function with the form as below

(32) $$\begin{array}{llll} \phi_{n}\left(x,t\right) & =\frac{D}{\gamma_{\text{0}}}[\gamma_{\text{1}}\text{ln}\left(x^{2}+K_{d}\right)+\gamma_{\text{2}}\text{arctan}\left(\frac{x}{\sqrt{K_{d}}}\right)+\gamma_{\text{3}}\text{ln}\left(Dn^{2}x^{2}-2mnx+\alpha \right)\; & & \;\\ & \;\;\;+\frac{\gamma_{\text{4}}}{\sqrt{D\alpha n^{2}-m^{2}n^{2}}}\text{arctan}\left(\frac{Dn^{2}x-mn}{\sqrt{D\alpha n^{2}-m^{2}n^{2}}}\right)\; & & \;\\ & \;\;\;+\frac{\gamma_{\text{5}}}{\sqrt{D\alpha n^{2}-m^{2}n^{2}}}\text{arctan}\left(\frac{Dn^{2}x-mn}{\sqrt{D\alpha n^{2}-m^{2}n^{2}}}\right)Acos\left(\Omega t\right)],\; & & \;\\ & \; & \end{array}$$

where *n, m, γ*_0_,*γ*_1_,*γ*_2_,*γ*_3 _and *γ*_4 _are given by Eq.(24). And

(33) $$\gamma_{\text{5}}=-2\alpha K_{d}Dn^{3}+n\alpha^{2}+4K_{d}n^{3}m^{2}+K_{d}n^{5}D^{2}.$$

Since the frequency *Ω *is very small, there is enough time for the system to reach the local equilibrium during the period of 1*/Ω. *On the other hand, assuming that the amplitude of input periodic signal is small enough (*A <<*1), it can not make the particles transit from a well to another well. Using the definition of MFPT and steepest descent method, one can obtain the expressions of transition rates *W_± _*out of *x_+_*, *x*_-_,

(34) $$W_{+}=\frac{\sqrt{\left|{U^{\prime \prime }}_{0}\left(x_{+}\right){U^{\prime \prime }}_{0}\left(x_{u}\right)\right|}}{2\pi }\text{exp}\left[\frac{\phi_{n}\left(x_{+},t\right)-\phi_{n}\left(x_{u},t\right)}{D}\right].$$

(35) $$W_{-}=\frac{\sqrt{\left|{U^{\prime \prime }}_{0}\left(x_{-}\right){U^{\prime \prime }}_{0}\left(x_{u}\right)\right|}}{2\pi }\text{exp}\left[\frac{\phi_{n}\left(x_{-},t\right)-\phi_{n}\left(x_{u},t\right)}{D}\right].$$

in which *U*(*x*), *x_+_*, *x*_-_, *x_u _*and *φ_n_*(*x, t*) are defined by Eq.(3) and Eq.(31), respectively.

For the general asymmetric nonlinear dynamical system, the SR phenomenon has been found, and the related theory has been developed [42]. Here, we only simply list this method for calculating signal to noise ratio (SNR).

The system is subjected to a time dependent signal *Acos(Ωt)*, up to first order on its amplitude (assumed to be small), the transition rates can be expanded as follows by two-state model theory:

(36) $$\begin{array}{c} W_{+}=\mu_{1}-\beta_{1}Acos\left(\Omega t\right),\\ W_{-}=\mu_{2}+\beta_{2}Acos\left(\Omega t\right). \end{array}$$

where the constants *μ*_1, 2 _and *β*_1, 2 _depend on the detailed structure of the system under study. For the asymmetric case, *μ*_1 _≠ *μ*_2 _and *β*_1 _= *β*_2_.

For the general asymmetric case we defined *R_S N R_*, the SNR, as the ratio of the strength of the output signal to the broadband noise output evaluated at the signal frequency. Finally, the expression of SNR is given by [42]

(37) $$R_{SNR}=\frac{A^{2}\pi {\text{(}\mu_{\text{1}}\beta_{2}+\mu_{\text{2}}\beta_{1})}^{2}}{\text{4}\mu_{\text{1}}\mu_{\text{2}}(\mu_{\text{1}}+\mu_{\text{2}})},$$

where

(38) $$\begin{array}{c} \mu_{1}=W_{+}{|}_{Acos\left(\Omega t\right)=0},\\ \mu_{2}=W_{-}{|}_{Acos\left(\Omega t\right)=0},\\ \beta_{1}={\left(\frac{dW_{+}}{d\left(Acos\left(\omega t\right)\right)}\right|}_{Acos\left(\Omega t\right)=0},\\ \beta_{2}={\left(\frac{dW_{-}}{d\left(Acos\left(\omega t\right)\right)}\right|}_{Acos\left(\Omega t\right)=0}, \end{array}$$

**Figure 8:**
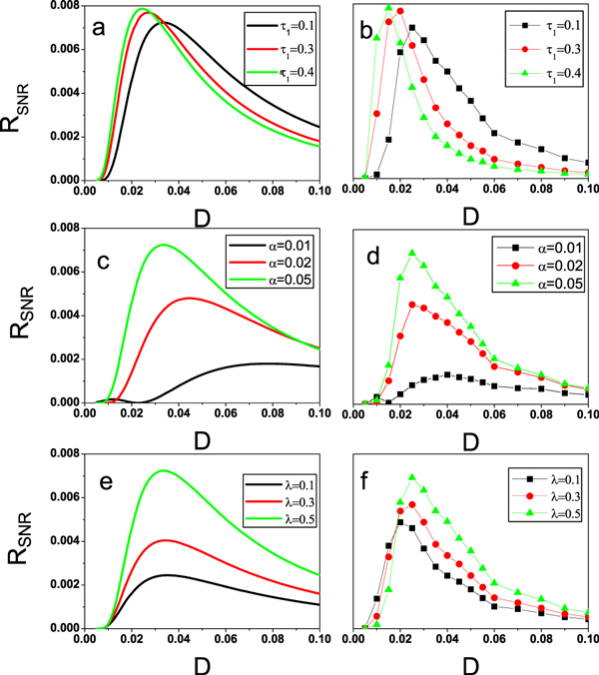
***R_S N R _*is plotted as a function of multiplicative noise intensity *D *with *A *= 0.08 and *Ω *= 0.001, the other parameter values are the same as those in Figure **2. The sold line on the left column represents the theoretical results (Eq.(31)), while the dot line on the right column is for the numerical results. From top to bottom, the results for different delay time with *α *= 0.01, *λ *= 0.5 in (a) and (b), for different additive noise intensity with *τ*_1 _= 0.1, *λ *= 0.5 in (c) and (d), for different correlated noise intensity with *τ*_1 _= 0.1, *α *= 0.015 in (e) and (f), are provided.

According to the expression of SNR in Eq.(37), the effects of the additive noise intensity *α*, the correlated noise intensity *λ *and the delay time *τ*_1 _on the SNR are analyzed. These results are plotted in Figures 8. In Figure 8, there exist one or two peaks which is the identifying characteristic of the SR phenomenon. It implicates that the noise-induced SR happens in this genetic regulatory system.

The SNR as a function of multiplicative noise intensity *D *with different delay time *τ*_1 _= 0.1, 0.3, 0.4 is plotted in Figure 8(a) according to the theoretical results (Eq.(31)) (the other parameters are fixed). It is found that there is a single peak in *R_SNR _*vs. *D. *The height of the peak is increased as the delay time *τ*_1 _increases, and the position of the peak shifts from the large *D *to small *D. *It implicates that the *R_SNR _*is enhanced with the increasement of delay time *τ*_1_. It must be pointed out that the observed SR is obvious when the additive noise intensity *α *is very weak.

The SNR as a function of the multiplicative noise intensity *D *with different additive noise intensity *α *= 0.01, 0.03, 0.05 is plotted in Figure 8(c) according to the theoretical results (Eq.(31)) (the other parameters are fixed). Comparing the curve of SNR for *α *= 0.01 to the curve of SNR for *α *= 0.02, the height of the peak is decreased greatly, and the position shifts slightly from the small value of *D *to the large value of *D. *Specially, when *α *= 0.05, the resonance bi-peaks structure is found in the curve of SNR. It means that the curve of SNR is changed from one peak to two peaks as *α *increases. It must be emphasized that the height of the first peak of SNR is more lower than the one of the second peak, and the position of the first peak is located in the very small value of the multiplicative noise intensity *D. *Namely, the additive noise intensity *α *can restrain the SR and induce the multiple SR.

The SNR as a function of the multiplicative noise intensity *D *with different correlated noise intensity *λ *= 0.1, 0.3, 0.5 is shown in Figure 8(e) according to the theoretical results (Eq.(31)) (the other parameters are fixed). It is seen that the height of the peak is enhanced greatly as the *λ *increases, the positions of the peaks are almost not distinct. It means that the correlated noise intensity *λ *can improve the SR.

Why these different control parameters exhibit various regulatory properties on the SR? One possible reason is that the potential function of the bistable gene model is adjusted differently. The symmetry of potential wells and the height of potential barrier have different dependences on these parameters. The quantitative analysis about the underlying mechanisms of time delay *-*enhance SR is our next task.

In order to check the valid of our theoretical approximate method, the numerical simulation is performed by directly integrating the Eq.(28) with Eqs.(5)-(8). Using the Euler method, the numerical data of time series are calculated using a fast Fourier transform. To reduce the variance of the result, the 1024 ensembles of power spectra are averaged. The output signal-to-noise ratio is defined as *R *= 10*log*10(*S_p_*(*Ω_s_*)*/S_n_*(*Ω_s_*)), where *S_p_*(*Ω_s_*) is the height of the peak in the power spectrum at the input frequency *Ω_s _*and *S_n_*(*Ω_s_*) is the height of the noisy background in the power spectrum around *Ω*_*s*_. The parameters are chosen as the same value in the theoretical analysis. The results are plotted in Figure 8(b), Figure 8(d) and Figure 8(f). Compared its to the theoretical results (Figure 8(a), Figure 8(c) and Figure 8(e)), respectively, it is clear that the trends of the approximate theoretical results in the SNR are consistent with the numerical simulation, which implies that the approximate method is credible.

### Results for case II

When the time delay appears in the Hill function, Eq.(10) becomes a nonlinear time delay stochastic equation. It is difficult to deal with the small time delay approximate method from the aspect of the theory. Hence the following results are given by direct simulation for the stochastic delay differential equation, i.e., Eq.(10), which can be formally integrated by using a simple forward Eular algorithm with a small time step for time delay.

The forward Euler algorithm with a small time step *Δt *can be formally integrated as

(39) $$\begin{array}{c} x\left(t+\Delta t\right)=x\left(t\right)+\left(\frac{k_{f}x^{2}\left(\tau_{\text{2}}\right)}{x^{2}\left(\tau_{\text{2}}\right)+K_{d}}-k_{d}x\left(t\right)+R_{bas}\right)\Delta t\\ \;\;-x\left(t\right)\sqrt{D\Delta t}N\left(t\right)+\lambda \sqrt{\alpha \Delta t}N\left(t\right)+\sqrt{\alpha \left(1-\lambda^{\text{2}}\right)}M\left(t\right). \end{array}$$

**Figure 9:**
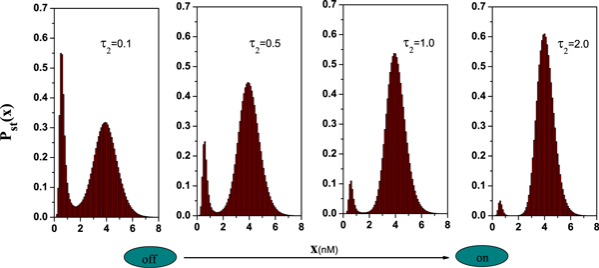
**The numerical simulations of the probability distribution *P_st_*(*x*) are plotted with the different delay time *τ*_2 _with *α *= 0.005, *D *= 0.15 and *λ *= 0.3. From left to right *τ*_2 _= 0.1, 0.5,1.0 and 2.0. The other parameter values are the same as those in Figure **2.

**Figure 10:**
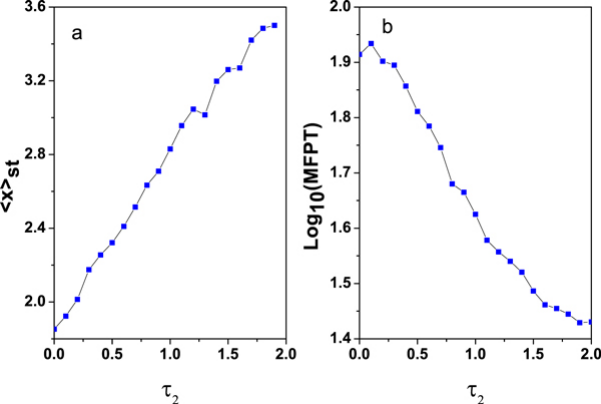
**The numerical simulations of (a) *< x >_st _*and (b) MFPT are plotted as a function of delay time *τ*_2_. *α *= 0.005, *D *= 0.03 and *λ *= 0.3. The other parameter values are the same as those in Figure **2.

Where $N\left(t\right)={\left[-4\text{ln}a\right]}^{\frac{1}{2}}cos\left(2\pi b\right),\;\;M\left(t\right)={\left[-4\text{ln}c\right]}^{\frac{1}{2}}cos\left(2\pi d\right)$ and *a*, *b*, *c*, *d *are all independent random numbers. The Box-Mueller algorithm is used to generate Gaussian white noise. Using Euler procedure, the time-discrete numerical data are calculated with the integration step of *Δt *= 0.001. An ensemble of *N *= 10^6 ^realizations of *x *is obtained from Eq.(10) by numerical calculations. For each realization of *x *the cycle is repeated for 1000 times. Accordingly, the stationary probability distribution *P_st_*(*x*) and the mean value (*x*)_*st *_can be obtained and shown in Figures 9-10. On the other hand, it must be pointed out that the range of time delay *τ*_2 _is unlimited. But in the case I the time delay *τ*_1 _is very small since the theoretical approximate method is only valid for small time delay *τ*_1_.

#### Steady-state probability distribution

Figure 9 shows the SPD as a function of the TF-A monomer concentration *x *for different delay time (the other parameters are fixed). The peak height of TF-A monomer concentration distribution near the low concentration state is higher than that near the high concentration state when the delay time is small. It implies that the gene switch is mainly in the "off" position by tuning the delay time to a very low value. However, if increasing the delay time, the peak height of TF-A monomer concentration distribution near the high concentration state becomes more pronounced. It means that the concentration of TF-A monomer increases, and a jump of the switch to the "on" position occurs. Therefore, delay time *τ*_2 _can be also used as a control parameter for the switch process in the genetic regulatory system. However, compared with case I, the time delay *τ*_1 _induces the transition of gene switch from "on" to "off".

#### Mean value

The numerical results of the mean value of *x*(*t*) for this system as a function of *τ*_2 _are plotted in Figure 10(a). The result presents the mean value of *x*(*t*) increases with *τ*_2 _increasing. In summary, when the model incorporated a nonlinear time delay $\tau^{{}^{\prime }}=\tau_{\text{1}}^{\text{'}}+\tau_{\text{2}}^{\text{'}}$, this delay time induces the switch from the "off" state to the "on" state. It is noticed that the time delays *τ*_2 _and *τ*_1 _play the opposite roles in our genetic regulatory process.

#### Mean first passage time

Similar, making use of the MFPT of the process *x*(*t*) to reach the high concentration state *x_+_*(*t*) with initial condition *x*(*t = *0) = *x*_-_, we can investigate the transition time from "on" state to "off" state. According to the definition of MFPT given by Hu [43], the MFPT as a function of *τ*_2 _is shown in Figure 10(b). It shows that MFPT decreases monotonously as *τ*_2 _increases. Physically, it means that the delay time *τ*_2 _can speed up the transition between the two steady states (low concentration state and high concentration state). Namely, the delay time can accelerate the transition of gene switch from "on" state to "off" state. The roles of *τ*_1 _and *τ*_2 _here are similar.

#### Stochastic resonance

**Figure 11:**
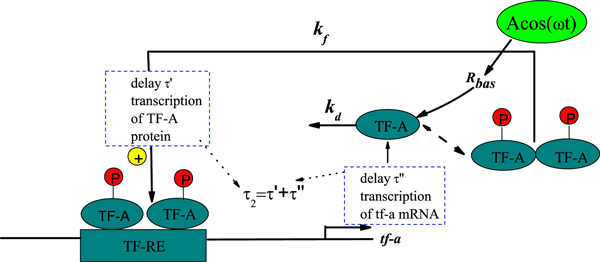
Model of genetic regulation with a positive autoregulatory feedback loop, an additive signal *Acos*(*Ωt*), and time delay *τ*_2 _= *τ' + τ″.*

Similar, we consider the gene transcriptional regulatory process subjected to a periodic signal *Acos*(*Ωt*), and the correlated noise and the time delay *τ*_2 _= *τ' + τ*″. The model is shown in Figure 11. Eq.(10) can be rewritten as,

(40) $$\frac{dx\left(t\right)}{dt}=\frac{k_{f}x^{2}\left(t-\tau_{\text{2}}\right)}{x^{2}\left(t-\tau_{\text{2}}\right)+K_{d}}-k_{d}x\left(t\right)+R_{bas}-x\left(t\right)\xi \left(t\right)+\eta \left(t\right)+Acos\left(\Omega t\right),$$

where *ξ*(*t*) and *η*(*t*) are the Gaussian white noise, and their statistical properties are given by Eqs.(5)-(8). *A *is the amplitude of input periodic signal, Ω is the frequency, and *τ*_2 _is the delay time.

**Figure 12:**
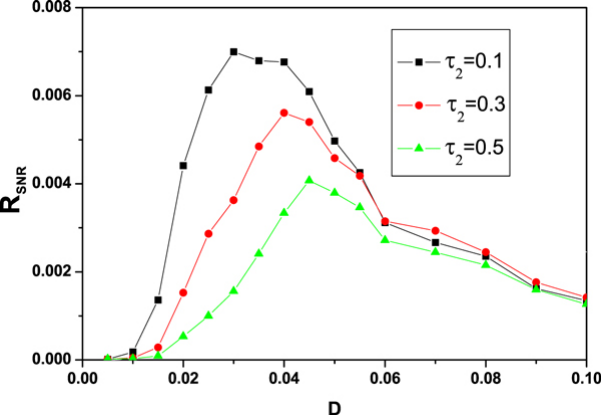
**Numerical simulation results of *R_S N R _*are plotted as the function of multiplicative noise intensity *D *for different delay time *τ*_2 _= 0.1, 0.3 and 0.5 with *α *= 0.015, *λ *= 0.3, *A *= 0.08 and Ω = 0.001, the other parameter values are the same as those in Figure **2.

Applying the numerical simulation method of calculating signal to noise ratio given by Ref. [6], we investigate the effects of the time delay *τ*_2 _on the SR. The SNR is defined as the ratio of the peak height of the power spectral intensity to the height of the noisy background at the same frequency. Figure 12 displays the SNR as a function of multiplicative noise intensity *D *with different delay time *τ*_2 _= 0.1, 0.3, 0.5, when the other parameters are fixed. It is found that there is a single peak in *R_SNR _*vs. *D. *The height of the peak is decreased as the delay time *τ*_2 _increases, and the position of the peak shifts from the small *D *to large *D. *It implicates that the *R_SNR _*is weaken with the increasement of delay time *τ*_2_. It should be noted that *τ*_2 _can restrain the SR to occur. Comparing Figure 12 with Figure 8(a), we found that the effects of *τ*_1 _and *τ*_2 _on the SR is different. *τ*_1 _can enhance the SR, but *τ*_2 _can weaken the SR.

## Conclusions

In this article, the regulatory properties of time delay on gene switch and stochastic resonance are systematically studied based on a bistable gene transcriptional regulatory model. This gene model is driven by the correlated noise and time delay simultaneously. Two cases, including linear time delay appearing in the degradation process (case I) and nonlinear time delay appearing in the synthesis process (case II) are considered, respectively. We mainly focus our research on two aspects, i.e., the dynamical switch characters (including the steady probability distribution, the mean value and the mean first passage time) and the stochastic resonance phenomenon.

For case I: Our theoretical results show that (i) the delay time *τ*_1 _resulting from the degradation process can induce the gene switch process, i.e., the TF-A monomer concentration *x *shifts from the high concentration state to the low concentration state ("on"→ "off"). Increasement of delay time *τ*_1 _can further speed up the transition from "on" to "off" state. (ii) The stochastic resonance can be enhanced by the time delay *τ*_1 _and the correlated noise intensity λ. However, the additive noise original from the synthesis rate *R_bas _*suppresses the stochastic resonance. It is very novel that the bi-peaks structure is found when *a *= 0.05. Through our stochastic delay dynamic approach, the critical physiological control parameters to which the behavior of special genetic regulatory systems is particularly sensitive are identified. Our theoretical results based on small-delay time-approximation approach are consistent with the numerical simulation, which implies that the approximate method is reliable.

For case II: Our numerical simulation results show that time delay *τ*_2 _can also induce the gene switch, while different from case I, the TF-A monomer concentration shifts from the low concentration state to the high concentration state ("off"→ "on"). The time delays in two cases play the opposite roles. With increasing the time delay *τ*_2_, the transition from "on" to "off" state can be further accelerated, which is similar to case I. Moreover, it is found that the stochastic resonance can be weaken by the time delay *τ*_2_. These insights on the combined effects of noises and time delay would be beneficial to understanding the basic mechanism of how living systems optimally facilitate to function under real environments.

The main result of our works is the time delays in both case I and case II induce gene switch, and the switch process can be further accelerated with increasing time delay. In order to demonstrate this theoretical result, an example is provided by using a biological system, i.e., the inducible lac genetic switch for Escherichia coli cells [44]. In Ref. [44], the switching of the lac operon from one phenotype to the other incorporates parameters, obtained from recently published in vivo single-molecule fluorescence experiments, has been investigated. It is found that anomalous sub-diffusion for macromolecules, as measured experimentally [44], can affect greatly the switch behavior. The authors predict an increase in the rebinding rate of transcription factor due to anomalous sub-diffusion. The underlying mechanism can be illustrated as below: the anomalous sub-diffusion behavior of the transcription factor causes it to spend more time (i.e., larger time delay) near the operator following unbinding than would be expected for purely Brownian diffusion, leading to more encounters with the operator and a potentially greater probability of rebinding. Hence this means that the time delay due to sub-diffusion in cellular crowding environment can increase the switch process of lac genetic system for Escherichia coli easily. It is consistent with our theoretical finding. Though a detailed modeling for sub-diffusion is not included in our work, the effect of complex sub-diffusion is replaced by introducing directly time delay. A full computational study of gene transcriptional system under macromolecule crowding using spatially resolved models is our next task.

To test our predictions quantitatively, one would ideally like to perform an experiment on this gene transcriptional regulatory model with tunable time delay and noise intensity, in which all the parameters concentrations of components and rate constants are the same as our theoretical model. To our knowledge, this clearly seems a very difficult experiment to perform, what we do is to give a primary picture of the integrated effects of time delay and noise. Recently, with the development of synthetic biology, some artificial gene networks are designed by genetic engineer. Moreover, it is increasingly being recognized that some biological parameters, including time delay and feedback strength, can be controlled by using micro-fluidic devices in gene regulatory network. So we wish that the time delay-accelerated transition of gene switch and time delay-enhanced or suppressed stochastic resonance could be examined in future.

## Competing interests

The authors declare that they have no competing interests.

## Authors' contributions

CJ Wang and M Yi conceived and designed the structure of this work. CJ Wang, KL Yang and LJ Yang performed the numerical experiments and analyzed the data. CJ Wang and M Yi wrote the paper. All authors have read and approved the final manuscript.
